# National surveys of radiofrequency field strengths from radio base stations in Africa

**DOI:** 10.1093/rpd/nct222

**Published:** 2013-09-17

**Authors:** Ken H. Joyner, Marthinus J. Van Wyk, Jack T. Rowley

**Affiliations:** 1 Joyner & Associates Pty Ltd, 34 Barrow Drive, Heathmont,VIC 3135, Australia; 2 EMSS Consulting, PO Box 1354, Stellenbosch 7599, South Africa; 3 GSM Association, 7th Floor, 5 New Street Square, London EC4A 3BF, UK

## Abstract

The authors analysed almost 260 000 measurement points from surveys of radiofrequency (RF) field strengths near radio base stations in seven African countries over two time frames from 2001 to 2003 and 2006 to 2012. The results of the national surveys were compared, chronological trends investigated and potential exposures compared by technology and with frequency modulation (FM) radio. The key findings from thes data are that irrespective of country, the year and mobile technology, RF fields at a ground level were only a small fraction of the international human RF exposure recommendations. Importantly, there has been no significant increase in typical measured levels since the introduction of 3G services. The mean levels in these African countries are similar to the reported levels for countries of Asia, Europe and North America using similar mobile technologies. The median level for the FM services in South Africa was comparable to the individual but generally lower than the combined mobile services.

## INTRODUCTION

In a previous publication two of the authors (J.R. and K.J.) assembled a very large database of over 173 000 radiofrequency (RF) field measurements around cellular radio base stations (RBS) from 23 countries across 5 continents^(1)^. The key findings were that irrespective of country, the year and cellular technology, potential exposures to radio signals at ground level were only a small fraction of the relevant human exposure standards and importantly, there had been no significant increase in potential exposure levels since the widespread introduction of 3G mobile services^(1)^.

In this publication another very large database of almost 260 000 measurements of cellular and FM radio signals from seven African countries has been investigated. After removal of noise data and other wireless broadband services the number of measurements available for analysis was 194 205, including the FM broadcast radio signals, over two time frames from 2001 to 03 and 2006 to 12. The wireless broadband services were only measured in South Africa and were excluded from the analysis because of the lack of comparable data from any of the other countries. In the case of South Africa, analogue terrestrial television (TV) is broadcast over a very wide frequency range (280 MHz to ∼800 MHz, with gaps in between). The situation became more complex with the commencement of digital TV services in the same bands, sometimes accompanied by temporary frequency allocations and changes as these services were introduced and for the planned switch over. Therefore, TV signals were not systematically measured in the surveys and the data are not included in this paper.

The aims of this current research project were to extend the earlier analysis to include a very large number of measurements from African countries to:

1. investigate similarities or differences between the results across the continent and between different technologies and frequency bands;
2. investigate any chronological trends in the potential exposure data where individual national surveys may have been conducted over a number of years;
3. compare the median levels of RF fields on the ground with accepted RF exposure limits for members of the general public and with the measured levels reported in our earlier study.

## RESULTS AND DISCUSSION

The data were sourced from surveys published in the open literature (Ghana and Nigeria), government sources (Ivory Coast, Mauritania and Zambia) and from independent experts conducting surveys (Botswana and South Africa). In the case of Botswana and South Africa the data were measured by staff of the organisation employing one of the authors (M.V.W.) and made available for this research with the permission of the network operators or governmental institutions.

The data were all measured using narrowband receivers which allowed the identification of frequency and power of individual signals transmitted from the cellular RBS. The usual approach is to identify and measure the control or pilot channel which is always transmitted at full power from the RBS^(1)^, although the South African and Botswana data also include traffic at the time of measurement.

The list of countries, years of measurement, mobile phone services and the number of measurements are given in Table 1. Table 2 contains the information that was available about the measurement equipment and techniques used in the national measurement surveys. Whilst all surveys used spectrum analysers there was minimal information about the actual set up for the analyser, such as, resolution bandwidth, sweep times and whether max or min hold was used.

**Table 1.** Countries from which data were sourced, the years over which the surveys were carried out, the types of services and the number of data points measured.

Country	Year of measurements	Individual cellular service and number of data points		Total data points per country	Reference
Botswana	2010	GSM900	181	543	(M.V.W.) Courtesy Botswana Communications Regulatory Authority
		GSM1800	181		
		WCDMA	181		
Ghana	2007	GSM900	50^a^	174	(2)
		GSM1800	50^a^		
	2010/11	GSM900	32		(3)
		GSM1800	42		
Ivory Coast	2009	GSM900	43	211	(4) and private communication^b^
		GSM1800	43		
	2010	GSM900	43		
		GSM1800	43		
		CDMA200	39		
Mauritania	2007	GSM900	146	899	(5)
		GSM1800	3		
		CDMA	204		
	2009	GSM900	108		
		GSM1800	12		
		CDMA	168		
	2010	GSM900	130		
		GSM1800	37		
		CDMA	258		
Nigeria	2001–03	GSM900/GSM1800	212^c^	212	(6)
South Africa^d^	2006	GSM900	21 016	188 148	(M.V.W.) Courtesy of the network operators
		GSM1800	16 758		
		WCDMA	14 673		
	2007	GSM900	20 494		
		GSM1800	15 771		
		WCDMA	14 821		
	2008	GSM900	11 181		
		GSM1800	7119		
		WCDMA	6358		
	2009	GSM900	6014		
		GSM1800	4097		
		WCDMA	3675		
	2010	GSM900	4614		
		GSM1800	2539		
		WCDMA	3062		
	2011	GSM900	3137		
		GSM1800	2441		
		WCDMA	2677		
	2012	GSM900	2589		
		GSM1800	1855		
		WCDMA	2677		
Zambia	2010	GSM900	126	315^e^	(7), Courtesy of the Zambia Information and Communications Technology Authority
		GSM1800	64		
		WCDMA2100	125		

^a^It was unclear how many individual measurements were made at GSM900 and GSM1800 but the authors have assumed that a measurement of each service was taken at each of the 50 base stations surveyed.

^b^Additional data for 2010 provided by Kouakou (private communication).

^c^Due to limited detail about the data set the authors were not able to differentiate the GSM900 and GSM1800 measurements but the authors have assumed that a measurement of each service was taken at each of the 106 base stations surveyed.

^d^The South African data were filtered to remove measurements that were within 10 dB of the noise floor for the instrumentation. A detailed discussion of this filtering has been included.

^e^The Zambia data were also filtered to remove data that were clearly noise floor measurements and this resulted in the exclusion of 63 data points predominantly from the GSM1800 measurements.

**Table 2.** Available information on measurement equipment and techniques used in the national measurement surveys.

Country	Spectrum analyzer			Antenna type	Measurement height	Measurement techniques and survey information
	Type	Settings				
		RBW	Max hold and frequency range			
Botswana	Refer ZA	Refer ZA	Refer ZA	Refer ZA	Refer ZA	Refer ZA
Ghana^(2, 3)^	Anritsu MS2601A^(2)^ and MS2721B^(3)^	NI^(2)^ or ^(3)^	NI^(2)^, 1–2 min, 0–2 GHz; 800–1000 MHz and 1700–1900 MHz^(3)^	Log –periodic^(2)^, Anritsu log-periodic MP666A^(3)^	1.5 m^(2)^, 1.7 m^(3)^	Six-minute weighted averaging time was used for all measurements. Measurements taken in direct line of sight to base station. The signals were measured during the day over a period of 3 h between 1000 and 1300 at a distance of ∼300 m from each base station^(2)^ Each measurement point consisted of three measured data based on the orientation of the antenna (horizontal, vertical and slanted) for duration of 1–2 min^(3)^
Ivory Coast^(4)^	NI	NI	NI	NI	NI	NI
Mauritania^(5)^	NI	NI	NI	NI	NI	Measurements performed mainly in the vicinity of the base station at distances ranging from 17 to 100 m on the three sectors
Nigeria^(6)^	Agilent E4407B ESA-E series	10 kHz and 30 kHz	Max hold on during entire scan time 940–960.1 MHz and 1.82–1.865 GHz	Horn	NI	Measurement of the base station signals conducted from 9:00 am to 5:00 pm local time. During this time interval the maximum hold button on the spectrum analyzer was enabled
South Africa	Narda SRM 3000 or 3006	1 MHz integration was performed using noise bandwidth=1.0552 RBW	30 s max hold 75 or 27 MHz to 3 GHz Frequency step size: 500 kHz	3-axis isotropic E-field antenna	1.5 m	At each site a number of positions were identified as positions of public interest and also positions of maximum potential exposure (where the main lobe hits the ground). A sweep of each identified area is performed to get the location of the local maximum. No positional sweeping was performed during the measurement itself
Zambia^(7)^	NI	Up to 20 MHz but no specific information	Max and min hold 80–2500 MHz; 920–960 MHz; 1.80–1.88 GHz and 2.11–2.17 GHz	Active antenna and PCD 8250 bi-conical	NI stationary measurements were made as well drive by tests	The stationary measurement consisted of fields from all angles in the *x*, *y* and *z* directions in a volume of 1 cubic metre of air at two distances (100 and 200 m) from the antenna. By using the instruments ‘max-hold’ function and by moving the antenna in the *x*, *y* and *z* directions for 6 min the maximum amplitude of the existing wavelengths was captured no matter of the polarisation Each spectral plot consists of 301 samples, often 10 MHz apart to cover the whole range of 0–3 GHz. Every sample consists of a min and max value detected by very fast peak detectors. When the next sweep commences the old data is updated with even stronger or weaker samples. After 10 sweeps the buffer contains the max and minima of the whole 10 sweeps for the frequency range. Storage is done every 0.9 seconds at the fastest rate, as the system normally is configured the rate is ∼1.1 s

NI, no information.

Using the procedures of IEC 62232:2011^(8)^ the expanded uncertainty of the South African measurements made using the Narda SRM-3000 was evaluated as 3.7 dB (confidence interval of 95 %), similar to that reported by others^(9)^. As variations in call traffic during a measurement can lead to up to 10 dB difference [if Global System for Mobile Communications (GSM) or Wideband Code Division Multiple Access (WCDMA) pilot channels only are present] a bias uncertainty of 6.27 dB for a total expanded uncertainty of 9.67 dB (including biased uncertainties, confidence interval of 95 %) was estimated. For the other surveys insufficient information was available to assess the uncertainty of the measurement equipment or approaches. As the authors noted in our earlier analysis of 23 countries there are many factors that can influence the accuracy of measurements; however, the only systematic difference found was that measurements with broadband equipment were higher than those using narrowband equipment^(1)^.

### Data treatment and filtering

**Figure 1.**
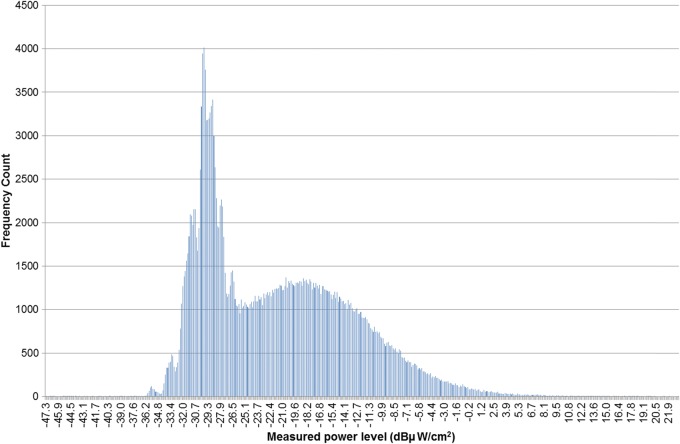
A histogram plot of the frequency of occurrence of particular power levels versus various measured power levels in dBµW cm^−2^ for the entire database of South African cellular measurements (188 148 points).

**Figure 2.**
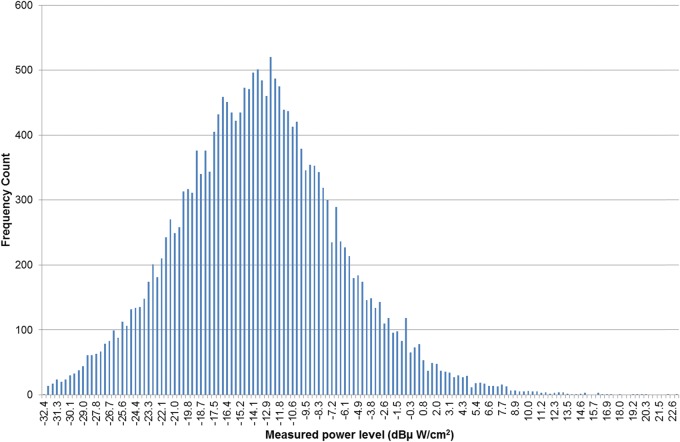
Histogram plot of the frequency of occurrence of particular power levels versus various measured power levels in dBµW cm^−2^ for GSM900 measurements for South African in 2006 (21 016 points).

Most of the analysis was focussed on the South African data because it was so extensive, both in years of measurement and number of measurements, and consequently it dominated the complete multinational data set. The authors also had the most complete information on the equipment used and measurement technique. The data also had the advantage of a generally consistent measurement methodology as the surveys were made by the same organisation and the authors had full access to the raw measurement data. Other researchers have reported that such environmental RF measurements may follow a normal^(10)^ or log–normal^(11, 12)^ distribution. However, when the log_10_ of the power density values of the South African data was plotted as a histogram (see Figure 1) a clear bimodal behaviour was apparent. This bimodal behaviour was unexpected and the cause was unknown. Interestingly, not all of the individual annual data sets exhibited the bimodal behaviour as evidenced by the histogram plot of the 900 MHz GSM log_10_ data for 2006 (Figure 2). The bimodal behaviour was present in each of the GSM1800 and WCDMA2100 data sets and only in two of the annual GSM900 data sets.

Prior to undertaking any statistical analysis and comparison with other national survey data it was essential that the individual distributions which comprised the bimodal distribution were separated and their source identified.

The separation of the two individual distributions from the bimodal distribution was performed using a Gaussian mixture model (GMM) probability distribution software package that returned the maximum likelihood estimation for the individual distributions. The GMM software package used is called scikits.learn.gmm^(13)^ and is implemented in the Python programming language^(14)^, in our case Python for Windows, and uses other sub-routines for multi-dimensional array and statistical analysis^(15)^.

Whilst each of the individual annual data sets per service was analysed with the GMM software the application to the consolidated South African data set provided most insight. The GMM analysis of the log_10_ of the consolidated data set (258 151 measurements) returned two approximately log–normal distributions, one of 99 648 data points with a median of −29.5 dBµW cm^−2^ and the other of 158 503 data points with a median of −17.3 dBµW cm^−2^.

Three possible explanations were postulated for the bimodal behaviour of the data:

1. The data were a mixture of line-of-sight (LOS) and non-line-of-sight (NLOS) measurements and the LOS was responsible for the higher median value distribution and the NLOS the lower median value distribution.
2. The survey procedure was a mixture of systematic and random measurements—the systematic measurements arose because the survey procedure required a search for the maximum values along a particular sector line leading to the higher median value distribution and the random nature arose because the measurements also included sites of local interest, possibly leading to the lower median value distribution.
3. The data set contained a significant Gaussian noise component which would account for the lower mean log–normal distribution.

It could be argued that these options could equally be expected to be present in the data sets from other researchers but they did not report such a bimodal behaviour^(11, 12)^. The authors were able to investigate options 1 and 2 through access to the raw data but the checks did not reveal any conclusive results; therefore, the possibility that the bimodal behaviour was indeed due to the presence of a Gaussian noise component was investigated.

The measurement equipment used for the South African surveys included a customised spectrum analyser. Both the Narda SRM 3000 and the later 3006 model were used for the measurements reported here. The measured data are captured from the spectrum analyser and stored in a specifically designed database package, Ixus [www.emssixus.com]. The South African data actually represented an integration per cellular service band (900, 1800, 2100 MHz and so on), that is, at each measurement position a frequency sweep was performed over each of the full cellular service bands and the received power was integrated across each of the operator's downlink licensed bands. Therefore, each measurement point represented an operator/technology combination, that is, multiple ‘services/operators’ were combined to arrive at the power density for each of the downlink bands. If there were no signals present in a service band at a particular measurement point then the integrated noise level across the service band would be recorded. In order to remove these noise values the authors decided to post-process the data to exclude readings that were within 10 dB of the noise floor. This technique is called noise suppression/threshold zeroing and is referred to in standards such as IEC 62232:2011^(8)^.

The following approach was used to filtering the South African data set:

1. The various spectrum data sets were stored with the integration result referred to above. The noise floor of the spectral components covering each service (such as GSM900) was obtained from the spectrum data, taking the lowest value sample in each service.
2. For each service, the integration of all the spectral components over the service is reported, but only taking spectral data points 10 dB or more above the noise floor into account. For many measurement positions a sufficient number of spectral components remained, mostly since at least one operator using this service was present at the measurement location. Some data points were completely removed through the filtering since no significant spectral components were present. This is especially true for newer technologies in the early years of the data set, since at that time many sites still only used earlier technologies, such as GSM900.
3. In total the data set for cellular services consisted of 248 472 data points collected at 82 824 positions. When filtered using the above procedure 60 324 data points were excluded (24.3 %) leaving 188 148 data points for GSM900, GSM1800 and WCDMA.

**Figure 3.**
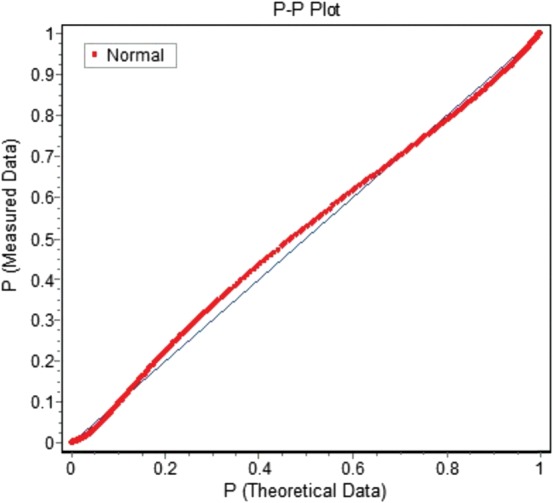
Probability–probability plot of a theoretical log–normal distribution versus distribution of the filtered measured data for South Africa. The closer to the straight line the more log–normal is the distribution.

The final South African data set of 188 148 data points (converted to dBµW cm^−2^) has been analysed to assess if it follows a log–normal distribution using a statistical distribution fitting software package^(16)^. The probability–probability plot of theoretical versus measured data in Figure 3 shows a fair agreement with log–normal, similar to that of Mann^(11)^. Fair agreement was confirmed by formal statistical tests showing a kurtosis value of 0.41256 and skewness of −0.26532 both of which should be near zero for a truly log–normal distribution.

## RESULTS

Table 3 summarises the main statistical parameters from Botswana, Ghana, Ivory Coast, Mauritania, Nigeria, South Africa and Zambia.

**Table 3.** Summary statistics for the measurement survey data by country, mobile technology and year.

Country	Period	Cellular technology	Number of measurements	Minimum (µW cm^−2^)	Maximum (µW cm^−2^)	Median (µW cm^−2^)	25th percentile (µW cm^−2^)	75th percentile (µW cm^−2^)
Botswana	2010	GSM900	181	1.71E−04	4.92E+00	4.54E−02	1.71E−02	1.15E−01
		GSM1800	181	9.20E−06	5.45E+00	2.73E−03	4.54E−04	1.02E−02
		WCDMA	181	3.28E−05	1.47E+00	2.76E−03	1.44E−04	1.10E−02
		2010 Totals	543	9.20E−06	5.45E+00	7.73E−03	1.03E−03	3.43E−02
		FM radio	181	6.94E−05	1.92E+01	1.52E−04	9.07E−05	1.78E−03
Ivory Coast^a^	2009	GSM900	43	3.90E−03	4.47E+00	3.42E−01	—	—
		GSM1800	43	3.32E−02	2.10E+01	1.17E+00	—	—
	2010	GSM900	43	2.48E−03	5.62E+00	7.94E−01	—	—
		GSM1800	43	1.31E−02	9.36E+00	4.35E−01	—	—
		CDMA850	39	2.15E−05	2.99E−01	5.61E−04	—	—
Ghana^b^	2007	GSM900	50	1.00E−06	1.00E−03	—	—	—
		GSM1800	50	1.00E−06	1.00E−02	—	—	—
	2010/2011	GSM900	32	8.50E−02	1.07E−01	—	—	—
		GSM1800	42	7.80E−02	1.19E−01	—	—	—
Mauritania	2007	GSM900	146	1.53E−06	1.43E−01	5.45E−03	1.10E−03	1.53E−02
		GSM1800	3	4.90E−06	9.14E−04	—	—	—
		CDMA	204	2.85E−03	2.51E−01	4.00E−02	1.64E−02	6.20E−02
		2007 Totals	353	1.53E−06	2.51E−01	1.97E−02	6.48E−03	4.67E−02
	2009	GSM900	108	7.65E−04	6.70E−01	7.68E−02	2.84E−02	1.51E−01
		GSM1800	12	1.12E−02	7.21E−02	2.86E−02	1.49E−02	6.37E−02
		CDMA	168	1.16E−03	5.25E−01	4.35E−02	1.64E−02	9.70E−02
		2099 Totals	288	7.65E−04	6.70E−01	5.27E−02	2.03E−02	1.19E−01
	2010	GSM900	130	8.68E−05	7.71E−01	2.66E−02	4.61E−03	1.01E−01
		GSM1800	37	6.37E−04	3.40E−01	1.55E−02	9.77E−03	7.76E−02
		CDMA	91	1.08E−04	1.30E−01	1.47E−02	4.37E−03	3.22E−02
		2010 Totals	258	8.68E−05	7.71E−01	2.05E−02	4.93E−03	6.02E−02
	2007–10	2007–10 Totals	899	1.53E−06	7.71E−01	2.87E−02	8.59E−03	6.68E−02
Nigeria^c^	2001–03	GSM900/GSM1800	212	—	5.94E−03	—	—	—
South Africa^d^	2006	GSM900	21 016	3.60E−05	3.90E+02	4.69E−02	1.62E−02	1.31E−01
		GSM1800	16 758	4.08E−05	9.94E+00	1.09E−02	2.44E−03	3.63E−02
		WCDMA	14 673	5.48E−05	1.34E+02	9.55E−03	3.33E−03	2.81E−02
		2006 Totals	52 447	3.60E−05	3.90E+02	1.93E−02	5.21E−03	6.48E−02
		FM radio	930	2.74E−04	2.88E+00	7.30E−03	1.85E−03	3.17E−02
	2007	GSM900	20 494	1.72E−05	1.50E+02	3.70E−02	1.15E−02	1.17E−01
		GSM1800	15 771	1.84E−05	6.33E+01	7.79E−03	1.87E−03	2.78E−02
		WCDMA	14 821	3.05E−05	2.47E+01	1.26E−02	3.92E−03	3.69E−02
		2007 Totals	51 086	1.72E−05	1.50E+02	1.69E−02	4.57E−03	5.86E−02
		FM radio	832	1.04E−04	4.52E+00	8.54E−03	1.98E−03	3.29E−02
	2008	GSM900	11 181	2.81E−05	1.58E+02	2.51E−02	6.25E−03	9.00E−02
		GSM1800	7119	2.35E−05	5.41E+01	7.97E−03	1.62E−03	3.01E−02
		WCDMA	6358	4.03E−05	9.64E+01	1.72E−02	4.97E−03	5.09E−02
		2008 Totals	24 658	2.35E−05	1.58E+02	1.66E−02	3.90E−03	5.72E−02
		FM radio	571	2.87E−04	3.25E+00	1.30E−02	2.74E−03	5.31E−02
	2009	GSM900	6014	1.67E−05	1.59E+01	2.70E−02	8.89E−03	7.42E−02
		GSM1800	4097	1.70E−05	2.25E+00	6.53E−03	1.57E−03	2.25E−02
		WCDMA	3675	3.87E−05	1.74E+01	1.80E−02	5.79E−03	4.88E−02
		2009 Totals	13 786	1.67E−05	1.74E+01	1.67E−02	4.63E−03	5.04E−02
		FM radio	219	2.80E−04	9.13E+00	9.57E−03	1.84E−03	6.20E−02
	2010	GSM900	4614	2.91E−05	4.77E+01	3.61E−02	1.17E−02	1.06E−01
		GSM1800	2539	2.32E−05	4.70E+00	8.39E−03	2.01E−03	2.78E−02
		WCDMA	3062	3.87E−05	1.16E+01	1.75E−02	5.53E−03	5.24E−02
		2010 Totals	10 215	2.32E−05	4.77E+01	2.08E−02	5.79E−03	6.59E−02
		FM radio	318	2.14E−04	1.88E+00	1.11E−02	1.80E−03	9.36E−02
	2011	GSM900	3137	2.16E−05	3.85E+01	5.66E−02	1.62E−02	1.56E−01
		GSM1800	2441	3.47E−06	5.69E+01	1.70E−02	2.71E−03	5.66E−02
		WCDMA	2677	2.85E−05	3.79E+01	2.82E−02	6.86E−03	8.21E−02
		2011 Totals	8255	3.47E−06	5.69E+01	3.22E−02	7.15E−03	9.64E−02
		FM radio	88	2.75E−04	2.49E+00	3.08E−03	8.91E−04	2.03E−02
	2012	GSM900	2589	2.13E−05	2.39E+02	6.67E−02	2.14E−02	1.97E−01
		GSM1800	1855	1.79E−05	8.14E+01	8.50E−03	1.33E−03	3.49E−02
		WCDMA	2078	3.29E−05	2.73E+01	2.19E−02	6.01E−03	6.63E−02
		2012 Totals	6522	1.79E−05	2.39E+02	2.78E−02	6.33E−03	9.72E−02
		FM radio	96	2.33E−04	1.76E+00	3.90E−03	1.26E−03	2.28E−02
	2006–12	GSM900	77 282	1.67E−05	3.90E+02	3.80E−02	1.18E−02	1.14E−01
		GSM1800	56 955	3.47E−06	8.14E+01	8.56E−03	1.89E−03	3.11E−02
		WCDMA	53 911	2.85E−05	1.34E+02	1.35E−02	4.21E−03	4.15E−02
		2006–12 Totals	188 148	3.47E−06	3.90E+02	1.85E−02	4.82E−03	6.26E−02
		FM radio	3562	2.95E−05	9.13E+00	7.49E−03	1.79E−03	3.70E−02
Zambia	2009	GSM 900	126	2.11E−04	5.29E−01	1.06E−02	4.20E−03	3.34E−02
		GSM1800	64	5.29E−07	1.33E−01	1.50E−03	3.34E−04	8.39E−03
		WCDMA	125	1.67E−04	1.06E−01	2.11E−04	1.67E−04	2.11E−04
		Totals	315	5.29E−07	5.29E−01	1.33E−03	2.11E−04	1.06E−02

^a^The authors did not have access to raw data for the Ivory Coast to calculate the 25th and 75th percentiles nor the total statistics but the medians per service and year were reported and presented here.

^b^The authors only had access to aggregated data and it was not possible to calculate the median, 25th and 75th percentiles for the annual data or the total statistics.

^c^For Nigeria the authors were only able to identify a maximum value of 5.94E−03 µW cm^−2^ for GSM900, the other values reported were averages and were very low but the data were not included in the table.

^d^For South Africa the statistical calculations were done on the filtered data after log-transformation and then converted back for presentation in the table.

**Figure 4.**
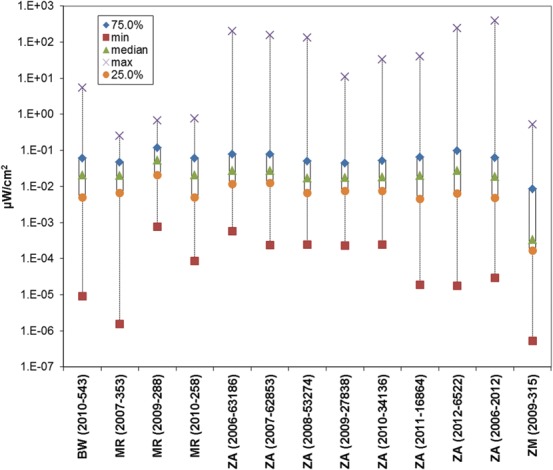
Plot of minimum (squares), maximum (cross), median (triangles), 25th (circles) and 75th (diamonds) percentiles for mobile services data from Mauritania (MR), South Africa (ZA) and Zambia (ZM) with units of µW cm^−2^. In brackets are the measurement year followed by the number of points, except for the last ZA entry, which is all mobile data 2006–12.

Similar to the approach taken by Manassas *et al*.^(10)^, Figure 4 is a plot of the maximum, minimum, median, 25th and 75th percentiles, which a visual inspection shows remain steady regardless of year and technology. The figure does not include Ghana, the Ivory Coast and Nigeria due to non-availability of percentile information. The results for this set of African countries are qualitatively and quantitatively similar to the findings of RF measurement surveys conducted in the Americas, Europe and Asia^(1)^ where the global weighted average was 0.073 µW cm^−2^. The mean for the filtered South African data set was 0.016 µW cm^−2^.

The instantaneous power flux density from a mobile base station varies throughout the day in response to call traffic. Some extrapolation methods recommend multiplying the short-term measured value by a scaling factor related to the total number of transmitters operating at the site; however, this may significantly overestimate realistic exposure^(17)^. Network-based monitoring of the downlink power distributions for GSM and 3G/WCDMA mobile networks reported that the 90th percentile during high traffic hours for GSM sites (with two or more transceivers installed) was 65 % of the maximum and 43 % of the maximum for 3G^(18)^. Therefore, the conservative extrapolation factor of 2.5 for GSM and 3G/WCDMA^(19)^ was used. Using this extrapolation factor the mean for the filtered South African data would become 0.040 μW cm^−2^. Further conservativeness is included in this extrapolated result since the South African data includes traffic at the time of measurement. The comparable levels would be expected as the cellular services are based on globally standardised technologies and should address a concern sometimes expressed about possible sub-standard network equipment or higher powers being used in developing markets.

Table 4 compares the statistical data of the resultant distributions after the full data set was treated using GMM analysis and the filtering of data within 10 dB of the noise floor. There are certainly some striking similarities between the quartiles, which would confirm that the original data set was impacted by noise. It also indicates that the GMM approach may be useful where it is not possible to access the original spectral measurements. A noise filter level of 10 dB was chosen, which still left an appreciable skew in the data set and guidance on an appropriate level may be worth discussion by international committees developing RF measurement standards such as IEC 62232:2011^(8)^. For comparison the difference between the sensitivity of the instrumentation and the minimum measured level from Joseph *et al*.^(12)^ was 1.94, 16.3, 4.86 and 5.26 dB for FM, GSM900, GSM1800 and UMTS signals, respectively. The consequence of too little or too much filtering will skew the distributions and their statistical parameters to lower or higher values, respectively.

**Table 4.** Comparison between the percentile data of the higher median value distribution as determined by the GMM approach and the percentile data for the filtered distribution.

Percentiles	Filtered data		GMM analysis (higher mean value distribution)	
Data points	188 148		158 503	
	dBµW cm^−2^	µW cm^−2^	dBµW cm^−2^	µW cm^−2^
Min	−54.59	3.48E−06	−47.30	1.88E−05
25 % (Q1)	−23.17	4.82E−03	−21.54	7.00E−03
50 % (Median)	−17.34	1.85E−02	−17.30	1.86E−02
75 % (Q3)	−12.03	6.27E−02	−12.50	5.65E−02
Max	25.91	3.90E+02	23.00	2.00E+02

**Figure 5.**
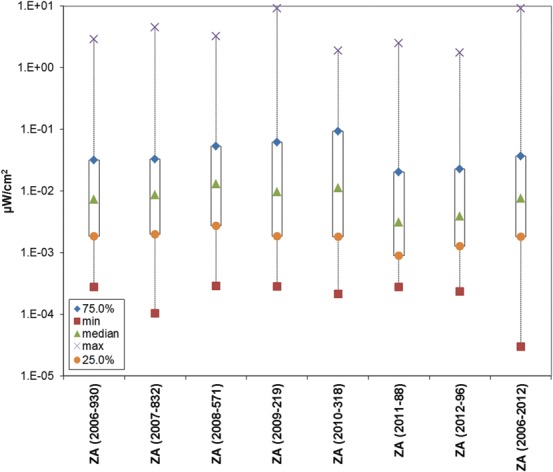
Plot of minimum (squares), maximum (cross), median (triangles), 25th (circles) and 75th (diamonds) percentiles for FM data from South Africa (ZA) with units of µW cm^−2^. In brackets are the measurement year followed by the number of points, except for the last entry which is for all FM data 2006–12.

During the surveys in South Africa it was routine to also measure the signal strengths of the FM radio services. These FM data were most affected by the noise floor filtering as compared with the cellular services. For the chosen measurement device attenuation, more than 95 % of the FM data points were removed during the noise filtering. A three-country RF survey (Belgium, the Netherlands and Sweden) in various living environments also found that cellular services were more likely to be above the measurement noise floor than FM broadcast^(12)^. In Figure 5 a plot of the maximum, minimum, median, 25th and 75th percentiles for the FM data for South Africa are presented. Points to note are as follows:

- The median value of 7.49E−03 µW cm^−2^ is higher than that reported by Joseph *et al.*^(12)^ for outdoor FM signals of 1.3E−03 µW cm^−2^ but lower than that reported by Lahham *et al*.^(20)^ of 0.148 µW cm^−2^.
- The median value of 7.49E−03 µW cm^−2^ for the FM services in South Africa is comparable with the individual mobile services but generally lower than the combined mobile services as shown in Table 4. Using the extrapolation factor of 2.5 for the mobile services the medians for GSM900, 1800 and WCDMA increase to 9.5E−2, 2.14E−2 and 3.38E−2, respectively, so that the mobile services are between about 3 and 12 times higher than FM.

The International Commission for Non-Ionising Radiation Protection (ICNIRP) has published, and confirmed as still valid, human exposure limits for members of the general public who are exposed to environmental levels of RF fields^(21, 22)^. The ICNIRP limit, median level per service for the South African data and the number of times below the ICNIRP limit are shown in Table 5. In Table 6 the findings were compared for the South African measurements of mobile services with the results from a 16-country pooled analysis^(1)^, France^(23)^ and recently published data for mobile services from a geographically diverse selection of countries: China^(24)^ (and the associated online Supplementary data ), Iran^(25)^, Saudi Arabia^(26)^ and the West Bank Palestine^(20)^. The spread of values is typical of the range observed in the larger set of 23 countries and relates to factors such as measurement equipment and choice of measurement location^(1)^. The results for South Africa are in the bottom half of the range of values in the table.

**Table 5.** The ICNIRP limit, median level per service for the filtered South African data and the number of times below the ICNIRP limit.

Service	ICNIRP limit for general public (µW cm^−2^)	Median measured level for the filtered South African data (µW cm^−2^)	Factor below ICNIRP limit
FM radio	200	7.49E−03	26 700
GSM900	450	3.80E−02	11 800
GSM1800	900	8.56E−03	105 100
WCDMA	1000	1.35E−02	74 000

**Table 6.** Comparison of measured levels for base stations from South Africa with a sample of recently reported measurements for other countries.

Country	Mobile services	µW cm^−2^
South Africa: mean	GSM900, GSM1800, 3G/WCDMA	0.016
16 countries: mean^a^	All Mobile	0.152
China: weighted mean^b^	GSM900	0.077
	GSM1800	0.046
France: means^c^	GSM900	0.112
	GSM100	0.047
	UMTS	0.014
Iran: median^d^	GSM900, GSM1800	0.014
Saudi Arabia: mean^e^	GSM900, GSM1800, 3G/WCDMA	0.061
West Bank, Palestine: mean	GSM900	0.089

^a^Austria, Australia, Belgium, Canada, Germany, Greece, Hungary, Ireland, Japan, Malaysia, Netherlands, South Korea, Spain, Sweden, Thailand, UK. For the mobile technologies see ref. (1).

^b^These values are the weighted means of the averages reported in ref. (24).

^c^These readings were from the period 2004 to 2007 and are the means for outdoor measurements in ref. (23).

^d^Median value reported for measurements near 28 hospitals in Tehran^(25)^. Mean not available.

^e^Calculated from the reported^(26)^ average exposure quotient of 0.0136 % and using the limit value of 450 µW cm^−2^.

## CONCLUSIONS

A very large database of measurements from seven countries from the African continent have been analysed and have shown that:

1. The signal strengths for the cellular bands are strikingly constant in both time and across countries despite the wide-scale introduction of 3G services.
2. It is important to establish the noise floor of the measurement system and to ensure that automated measurement systems are indeed recording actual signals rather than noise. The effect of noise is to skew statistical parameters below actual values.
3. The distribution of environmental RF measurements is generally log normal.
4. FM signal strengths are relatively constant with time when there are several hundred data points included in the annual data set.
5. The median value for the combined FM services in South Africa is comparable to the individual mobile services but generally lower than the aggregate of all of the mobile services.
6. The median RF field levels at ground level for the combined data for the South African database are between 11 800 and 105 100 times below the respective limits for the general public.

Measured environmental RF signal levels from mobile network antenna sites in African countries are typically many thousands of times below the international human RF exposure recommendations, similar to values reported in countries of the Americas. Europe and Asia, and the consensus of international public health authorities is that these weak signals do not cause adverse health effects^(27)^.

## FUNDING

This work was supported by the GSM Association [K.J. and J.R.]; EMSS has received payments from operators and regulatory authorities for conducting RF measurement surveys [M.V.W.].

## CONFLICT OF INTEREST

The views are solely those of the authors.
